# The Role of Childhood Trauma in Psychosis and Schizophrenia: A Systematic Review

**DOI:** 10.7759/cureus.21466

**Published:** 2022-01-21

**Authors:** Bithaiah Inyang, Faisal J Gondal, Godwin A Abah, Mahesh Minnal Dhandapani, Manasa Manne, Manish Khanna, Sabitha Challa, Ahmed S Kabeil, Lubna Mohammed

**Affiliations:** 1 Research, California Institute of Behavioral Neurosciences & Psychology, Fairfield, USA; 2 Internal Medicine, California Institute of Behavioral Neurosciences & Psychology, Fairfield, USA; 3 Family Medicine, California Institute of Behavioral Neurosciences & Psychology, Fairfield, USA

**Keywords:** childhood maltreatment, childhood adversity, psychotic disorder, adverse life events, psychosis, schizophrenia, schizophrenia spectrum disorders, child abuse and neglect, childhood trauma, first episode psychosis

## Abstract

Schizophrenia (SCZ) is a prevalent cause of disability worldwide. Distinguished mainly by psychosis, behavioral alterations could range from hallucinations to delusions. This systematic review examines evidence of a relationship between childhood trauma/adverse life events and psychosis, especially in SCZ. A methodical search provided reproducible results using these five databases: PubMed, ScienceDirect, Semantic Scholar, JSTOR, and Cochrane Library. The systematic search focused on articles published between July 2016 and July 2021. The search strategy utilized specific keywords relevant to SCZ, psychosis, and childhood trauma. The formulation of specified inclusion and exclusion criteria was necessary to ensure a comprehensive narrowed-down search, such as the inclusion of free full-text articles published or translated in English and exclusion of irrelevant subject areas. Using the Preferred Reporting Items for Systematic Reviews and Meta-Analyses (PRISMA) guidelines, a strategic search initially identified 741 articles; three additional articles were identified from citation searching. After relevance screening, duplicate removal, and quality appraisal, 12 studies from databases/registers and three from citation searching met the criteria proving relevance to our review with minimal evidence of bias. The final selected 15 studies included observational studies and reviews. A review of relevant data unveiled findings on childhood adversity, individual lived experiences, and their involvement in SCZ. Evidence suggests that certain neurobiological processes occur in brain after trauma. The inflammation and dysregulation from oxidative stress predispose patients to an at-risk-mental state, facilitating the progression to SCZ. This review encourages further evaluation of early trauma detection and the potential benefits of early intervention.

## Introduction and background

Schizophrenia (SCZ) affects 20 million people worldwide, and it is one of the top 15 leading causes of disability. SCZ typically presents with positive, negative, and cognitive symptoms. Auditory hallucinations and thought disorders are consistent with positive symptoms. These symptoms usually respond favorably to antipsychotic treatment in most individuals. On the contrary, negative symptoms such as social withdrawal and flat affect, and cognitive symptoms including learning and attention disorders pose significant resistance to available therapy modalities [1].

Based on the dopamine hypothesis, positive symptoms are related to the hyperfunctionality of dopamine D2 receptor neurotransmission in limbic and subcortical brain regions. Hypoactivity of D1 receptor neurotransmission contributes to negative and cognitive symptoms of SCZ [2]. An alteration in dopamine function is strongly associated with the onset of psychotic symptoms. Increased subcortical dopamine production and release are predictable for positive SCZ findings and treatment success [3]. Although the incidence of SCZ is higher in family members of affected individuals, there are theories of several natural surrounding factors in the development of SCZ. The involvement of these exposures favors the neurodevelopmental hypothesis of SCZ, suggesting that SCZ arises from both heritable traits and environmental exposures occurring throughout development from the prenatal period well onto adolescence [4].

Psychosis is a range of behavioral alterations related to a loss of connection with reality and insight. Psychosis is closely associated with SCZ; individuals with psychosis present with hallucinations and delusions. An altered connection within the thalamocortical pathway, specifically with the hippocampus, may cause an impediment to auditory processing. The prefrontal cortex (PFC) overactivation and deactivation of the striatal/thalamic network is closely associated with delusions. This interwoven relationship between these brain regions is crucial in understanding the complexity of psychotic disorders [3].

Childhood trauma (ChT), also known as childhood adversity, defines stressful life events such as physical, sexual, emotional abuse, and neglect [5,6]. In early developmental years, adverse childhood experiences (ACEs) comprise exposure to long-term environmental stressors such as childhood maltreatment, domestic violence, living in a household with ongoing substance abuse, and interpersonal loss [7]. Interpersonal loss entails experiencing parental death, divorce, or mental illness early in life before age 17 [8].

The Childhood Trauma Questionnaire (CTQ) is a valuable survey tool used to screen for experiences of abuse and neglect [9]. Growing evidence links ChT with inflammation, proposing that the pathophysiology of trauma-related psychopathology can be explained by inflammation [10]. Although psychotic symptoms typically begin in the age of 18-25 years, those who later develop SCZ have earlier cognitive deficits in childhood, suggesting that these cognitive deficits are an indicator of abnormal neurodevelopment, particularly when considering early developmental adversities [11].

Establishing the role of childhood adverse life events in psychotic disorders is the first step in anticipating and eventually mitigating the adulthood development of these life-altering disorders. This systematic review aims to summarize the effects of ChT in the development of SCZ/psychosis and discuss more specific details of this concept.

## Review

Methods

A systematic search in July 2021 was done using the following databases: PubMed, ScienceDirect, Semantic Scholar, JSTOR, and Cochrane Library. The search included investigations from the last five years (2016-2021), full free-text articles, published materials, subjects, and fields relevant to schizophrenia, psychosis, and childhood trauma. Other than PubMed, all database and registry searches included the following keywords: "Childhood trauma and Schizophrenia and Psychosis." On PubMed, the search strategy included a Medical Subject Headings (MeSH) combination derived from MEDLINE and individual keywords as shown below:

(Schizophrenia OR Psychotic disorder OR Psychosis AND Childhood trauma OR Child abuse OR Childhood adversity OR Childhood maltreatment OR Adverse life events AND "Schizophrenia/analysis"[Majr] OR "Schizophrenia/diagnosis"[Majr] OR "Schizophrenia/economics"[Majr] OR "Schizophrenia/epidemiology"[Majr] OR "Schizophrenia/ethnology"[Majr] OR "Schizophrenia/etiology"[Majr] OR "Schizophrenia/genetics"[Majr] OR "Schizophrenia/prevention and control"[Majr] OR "Schizophrenia/psychology"[Majr] OR "Schizophrenia/statistics and numerical data"[Majr] "Schizophrenia Spectrum and Other Psychotic Disorders/analysis"[Majr] OR "Schizophrenia Spectrum and Other Psychotic Disorders/diagnosis"[Majr] OR "Schizophrenia Spectrum and Other Psychotic Disorders/economics"[Majr] OR "Schizophrenia Spectrum and Other Psychotic Disorders/epidemiology"[Majr] OR "Schizophrenia Spectrum and Other Psychotic Disorders/ethnology"[Majr] OR "Schizophrenia Spectrum and Other Psychotic Disorders/etiology"[Majr] OR "Schizophrenia Spectrum and Other Psychotic Disorders/genetics"[Majr] OR "Schizophrenia Spectrum and Other Psychotic Disorders/prevention and control"[Majr] OR "Schizophrenia Spectrum and Other Psychotic Disorders/psychology"[Majr] OR "Schizophrenia Spectrum and Other Psychotic Disorders/statistics and numerical data"[Majr] OR "Schizophrenia Spectrum and Other Psychotic Disorders/transmission"[Majr] AND "Psychotic Disorders/analysis"[Majr] OR "Psychotic Disorders/diagnosis"[Majr] OR "Psychotic Disorders/economics"[Majr] OR "Psychotic Disorders/epidemiology"[Majr] OR "Psychotic Disorders/ethnology"[Majr] OR "Psychotic Disorders/etiology"[Majr] OR "Psychotic Disorders/genetics"[Majr] OR "Psychotic Disorders/prevention and control"[Majr] OR "Psychotic Disorders/psychology"[Majr] OR "Psychotic Disorders/statistics and numerical data"[Majr] AND "Adult Survivors of Child Adverse Events/psychology"[Majr] OR "Adult Survivors of Child Adverse Events/statistics and numerical data"[Majr] AND "Child Abuse/complications"[Majr] OR "Child Abuse/economics"[Majr] OR "Child Abuse/epidemiology"[Majr] OR "Child Abuse/ethnology"[Majr] OR "Child Abuse/psychology"[Majr]")

This search strategy ensured that recent and pertinent articles moved on for further evaluation. Inclusion criteria included published articles within the last five years (2016-2021), free full-text articles, reviews, research, journals, studies, clinical trials, meta-analysis, and case reports. Fields of study related to medicine and psychology were included. Exclusion criteria considered the exclusion of the following subject areas: pharmacology, toxicology, and pharmaceutical science, immunology and microbiology, computer science, arts, and humanities. Book chapters and articles with unrelated confounding factors were also excluded.

Results

The search identified a total of 741 articles from databases and registers with the aid of filters using the inclusion and exclusion criteria noted above. EndNote assisted with removing duplicated articles identified from PubMed, JSTOR, and ScienceDirect. Then, a manual comparison of search outcomes on Semantic Scholar, Cochrane Library, citation searching, and OpenGrey (a grey literature registry) against residual articles on EndNote was done. In addition to the 741 articles, three additional articles were identified from citation searching.

After duplicate removal, 226 articles from databases/registers were screened manually for significance to our research topic. A total of 34 articles from databases/registers and the three articles from citation searching were sought for retrieval; 33 articles from databases/registers and three articles from citation searching were retrieved and sent for quality appraisal. Quality assessment tools Newcastle-Ottawa scale, Scale for the Assessment of Narrative Review Articles (SANRA) checklist, and Assessment of Multiple Systematic Reviews (AMSTAR) checklist for observational studies, literature reviews, and systematic/meta-analysis reviews, respectively, were used. The pass mark for articles considered in this review was a score of 70% or above.

**Figure 1:**
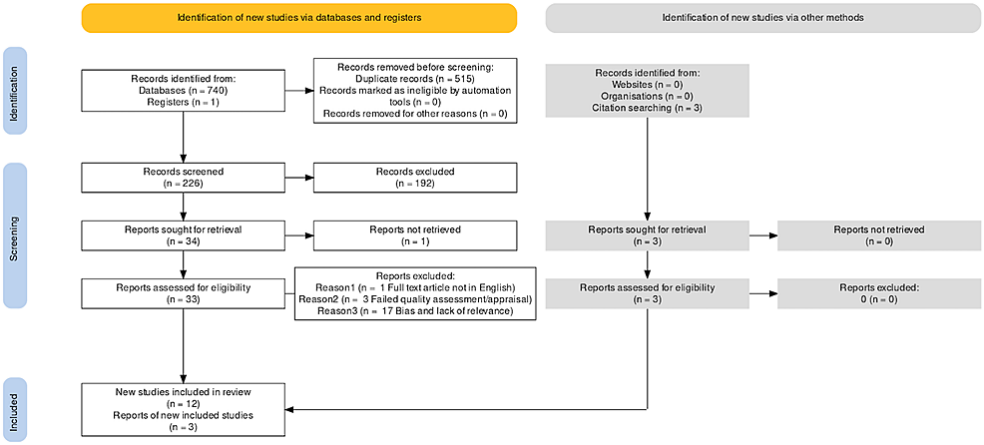
PRISMA 2020 flowchart PRISMA, Preferred Reporting Items for Systematic Reviews and Meta-Analyses

Two individuals conducted the quality assessment; the second author verified the results noted by the first author. Finally, 12 studies from databases/registers and three from citation searching were identified to pass the quality check, relevant to the research topic, and contained minimum to no bias. The identification process breakdown is shown in the Preferred Reporting Items for Systematic Reviews and Meta-Analyses (PRISMA) flow chart (Figure 1) [12]. Table 1 analyses the focus, methods, and conclusions of the selected 15 studies.

**Table 1:** Analysis of selected studies reviewing the influence of trauma in the development of schizophrenia and psychosis BDNF: brain-derived neurotrophic factor, ChT: childhood trauma, CTQSF: Childhood Trauma Questionnaire Short Form, PANSS: Positive and Negative Syndrome Scale, SANRA: Scale for The Assessment of Narrative Review Articles, N/A: not applicable, SCZ: schizophrenia, PTSD: post-traumatic stress disorder, CTQ: Childhood Trauma Questionnaire, TEI: Traumatic Events Inventory, CAPS: Clinician-Administered PTSD Scale, MINI: Mini International Neuropsychiatric Interview, UHR: ultra high risk, SCID: Structured Clinical Interview for DSM-IV, DSM: Diagnostic and Statistical Manual of Mental Disorders, PAS: Premorbid Adjustment Scale, NES: Neurological Evaluation Scale, DI-PAD: Diagnostic Interview for Psychosis and Affective Disorders, LSAS: Liebowitz Social Anxiety Scale, Y-BOCS: Yale-Brown Obsessions and Compulsions Scale, PaSI: Panic and Schizophrenia Interview, ECR-RS: Experience in Close Relationships – Relationship Structures, ETISR-SF: Early Trauma Inventory Self Report – Short Form, ALSPAC: Avon Longitudinal Study of Parents and Children, PGS: polygenic scores, MRI: magnetic resonance imaging

	Study title	Author	Year	Type of study	Methods	Conclusion
1.	Types, prevalence and gender differences of childhood trauma in first-episode psychosis. What is the evidence that childhood trauma is related to symptoms and functional outcomes in first episode psychosis? A systematic review.	Vila-Badia et al. [13]	2021	Review	Systematic data search using three databases; Medline, PsycInfo, and Scopus.	Patients with first-episode psychosis have a high prevalence of ChT. There is a strong link between the increased frequency of ChT and the development of delusions and hallucinations.
2.	The relationship between childhood trauma and schizophrenia in the Genomics of Schizophrenia in the Xhosa people (SAX) study in South Africa.	Mall et al. [14]	2020	Observational study	SCZ patients and controls were recruited from healthcare facilities in the South African Western and Eastern Cape regions.	ChT is a vital predictor for psychosis and SCZ especially in low- and middle-income countries.
3.	The role of the interaction between the FKBP5 gene and stressful life events in the pathophysiology of schizophrenia: a narrative review.	Stramecki et al. [15]	2020	Review	N/A	FKBP5 gene plays a role in the evolution of psychosis and the progression of SCZ in response to chronic and acute stress and alteration of brain regions related to stress hormones.
4.	Genetic liability to schizophrenia is associated with exposure to traumatic events in childhood.	Sallis et al. [16]	2020	Observational study	ALSPAC was used to recruit all pregnant women within a range of due dates. The Norwegian Mother, Father, and Child Cohort Study (MoBa) was used to derive SCZ PGS and measures of trauma exposure.	There is evidence of a link between the SCZ PGS and most trauma subtypes investigated, except for bullying.
5.	Child maltreatment and psychosis.	Kaufman and Torbey [17]	2019	Review	N/A	There is substantial supporting evidence that patients with psychotic disorders and histories of child maltreatment have different clinical characteristics and treatment needs that separate them from patients without a history of child abuse.
6.	Childhood trauma in schizophrenia: current findings and research perspectives.	Popovic et al. [18]	2019	Review	N/A	ChT exposure results in the aberrant function of parietal areas involving memory and visual cortical areas linked to attention. Decreased connectivity in the amygdala in patients with increased exposure to childhood physical neglect and sexual abuse suggests that disruptions in specific brain networks affect cognitive abilities.
7.	Early trauma, attachment experiences, and comorbidities in schizophrenia.	Gabínio et al. [19]	2018	Observational study	Schizophrenic patients were diagnosed based on DSM 5 criteria in Campo Grande, Brazil. The study used the following assessment tools: DI-PAD, LSAS, Y-BOCS, PaSI, ECR-RS, and ETISR-SF.	Increased frequencies of cumulative early traumatic events prognosticate SCZ later in life. Panic and anxiety symptoms are predominant in children with unstable childhood emotional lives due to psychopathology vulnerability.
8.	Childhood trauma interacted with BDNF Val66Met influence schizophrenic symptoms.	Bi et al. [20]	2018	Observational study	The Chinese version of CTQSF and PANSS evaluated Chinese childhood abuse in selected schizophrenic patients.	BDNF Val66Met polymorphism predicts clinical schizophrenic symptoms, and BDNF Met/Met carriers show an increased risk of SCZ. The study noted that childhood trauma is probably the most important environmental factor associated with SCZ.
9.	A network approach to psychosis: pathways between childhood trauma and psychotic symptoms.	Isvoranu et al. [21]	2017	Observational study	Patients from 36 mental healthcare institutions in the Netherlands and Belgium meeting specific inclusion criteria were recruited. Specific networks were established using the Dutch version of CTQSF with PANSS symptoms.	This novel alternative approach to psychopathology conceptualizes mental disorders as causal systems of interacting symptoms. It suggests that several general psychopathology symptoms mediate the relationship between trauma and psychosis.
10.	Factors moderating the relationship between childhood trauma and premorbid adjustment in first-episode schizophrenia.	Kilian et al. [22]	2017	Observational study	SCID, CTQ Short Form, PAS, and NES were utilized to evaluate patients who met the inclusion criteria from the greater Cape Town area.	There is a complex association between risk factors, ChT, and premorbid adjustment backing pathways directly linked to psychosis.
11.	Adversity in childhood linked to elevated striatal dopamine function in adulthood.	Egerton et al. [23]	2016	Observational study	The study assesses childhood adversity using the Childhood Experience of Care and Abuse questionnaire, recruiting individuals from the same geographic area in South London, employing 18F-DOPA positron emission tomography.	Evidence presents that childhood adversity has a connection to elevated adulthood striatal dopamine function. Participants with severe physical or sexual abuse experienced in childhood have a significantly higher striatum dopamine function than those who had not.
12.	Childhood trauma, PTSD, and psychosis: findings from a highly traumatized, minority sample.	Powers et al. [24]	2016	Observational study	CTQ, TEI, CAPS, and the MINI, were used on participants with PTSD risk factors in a low socioeconomic, urban minority population in Atlanta, Georgia.	Compared to patients without a current psychotic disorder, those with current psychotic disorders had higher exposure to moderate-to-severe childhood abuse.
13.	Are specific early-life adversities associated with specific symptoms of psychosis? A patient study considering just world beliefs as a mediator.	Wickham and Bentall [25]	2016	Observational study	SCZ patients were recruited from North West England/North Wales and assessed using the following measures; PANSS, CTQ, Retrospective Bullying Questionnaire, the General Beliefs in a Just World Scale, and the Personal Belief in a Just World Scale.	Cognitive processes are essential determinants of childhood adverse life events, using the framework of mental patterns of psychosis. Hallucinations and paranoia can indicate various early life experiences.
14.	Childhood trauma and psychotic disorders: a systematic, critical review of the evidence.	Bendall et al. [26]	2008	Critical review	Initial search included three databases using specific inclusion and exclusion criteria.	Good control groups are needed to establish a connection between ChT and psychosis. Further research is available to clarify and analyse variables that mediate the correlation between ChT and psychosis, like substance abuse, family environment, and educational attainment.
15.	Trauma and construction of self and others following psychotic experiences.	Sporle [27]	2007	Review/thesis	The sample size was studied using assessment scales and repertory grids.	Experiencing severe childhood trauma conflicts with individual self-concept; the result mainly presents the primary trauma variable as sexual abuse. The consideration of trauma histories is notable with individuals who have experienced psychosis.

Discussion

Recent studies have demonstrated that individuals with SCZ are 2.7 times more likely to have experiences of ACEs than healthy controls [9]. This systematic review focuses on analysing the current data on ChT, psychosis, and SCZ, comparing individual findings to their interwoven relationships. This section will first address ChT broadly, including assessment tools for trauma identifying and trauma severity scaling. We will further concentrate on the different mechanisms involved in the relationship between SCZ, psychosis, and ACEs, including specific psychological outcomes, genetic findings, neural pathways, and brain modifications.

Exploring Facets of Childhood Trauma

The capability of a caregiver to provide a safe and healthy environment is directly proportional to the quality of a child's attachment style. A child's relationship with the caregiver plays a vital role in early attachment style formation. Children with responsive caregivers develop secure attachments and are more open to seeking support when faced with difficulties. On the other hand, children with unreliable caregivers that fail to care for their needs tend to develop an avoidant and resistant attachment style, learning to be emotionally self-reliant at an early age [19].

**Figure 2:**
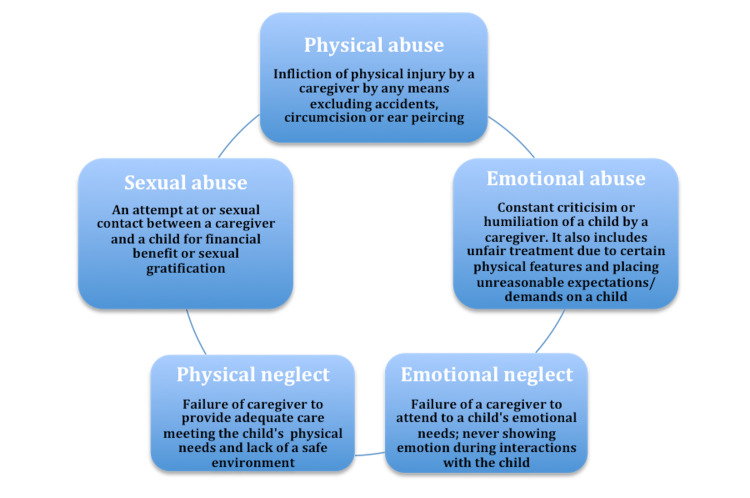
Classification of childhood trauma Here, a caregiver refers to any responsible adult with a relationship with or in a place of authority over a child.

Around a fourth of children encounter child abuse or neglect in their lifetime. It is crucial to highlight that 78% are neglect, 18% physical abuse, and 9% sexual abuse cases. The fatality rate for child maltreatment is significant annually, being the second leading cause of death in children less than one year of age. Exposure to violence can have lifelong health consequences during childhood, including poor emotional, physical, and mental health. The key to preventing these poor outcomes lies in preventing, adequately diagnosing, and treating all forms of child abuse. The goal is to decrease undetected and unreported cases by clinicians. The five types of childhood trauma addressed in this review are physical abuse, emotional abuse, sexual abuse, physical neglect, and emotional neglect [13,28]. Figure 2 provides a brief description of the different types of ChT, highlighting particular behaviors and patterns that caregivers can use to cause trauma [29,30].

Effects of Childhood Trauma on the Developing Brain

ChT is probably the most important environmental factor associated with SCZ [20]. There is evidence of multiple paths between traumatic experiences and psychosis. Mental disorders are systems of interacting symptoms through a framework of networks [21]. Psychotic symptoms in patients with a history of child abuse are more severe, persistent, and refractory to treatment [17]. In developmental years, exposure to neglect and abuse reveals severe adverse effects on the average neurobiological growth, leading to vulnerable neurobiology preceding disadvantageous psychiatric outcomes. Importantly, post-traumatic stress disorder (PTSD) and ACEs have a modifying effect on psychotic symptoms. Independent of ChT, patients with PTSD are five times more likely to be diagnosed with psychotic disorders than individuals without PTSD [24].

ChT is a form of severe stress that puts individuals in vulnerable mental states with the likelihood of developing mental disabilities such as SCZ. Multiple studies indicate that ACEs multiply the risk of developing psychosis and SCZ. In ultra-high-risk (UHR) individuals, higher levels of ChT result in more heightened positive, general, and depressive symptoms and poorer levels of global functioning. Histories of emotional neglect, physical neglect, emotional abuse, and sexual abuse are frequent in individuals at UHR. It is worth noting that female patients reporting episodes of physical abuse during childhood years have more psychotic and depressive symptoms when evaluated than their counterparts without a history of ChT [18].

Meta-analysis in individuals with psychotic disorders proposes a three-fold likelihood of having experienced some childhood adversity compared to healthy controls [23]. The relationship between trauma exposure and psychosis is heavily dependent on the onset, time frame, and nature of trauma. The risk of developing psychopathology is exceptionally high in home environments with recurrent exposure to ongoing traumatic events [24]. The relationship between exposure to ChT and SCZ is strongly dose-responsive as noted above, concluding that diagnosis is most feasible in individuals with multiple exposures to ACEs. A recent study in South Africa made a similar discovery when exploring the relationship between childhood trauma and SCZ. One experience of physical or emotional trauma had no significant association with SCZ; still, in individuals with greater than two experiences of these traumas, there is an increase in the odds of developing SCZ, suggesting that physical and emotional childhood adversity, when occurring infrequently, does not necessarily increase the risk of SCZ. On the other hand, one experience of sexual abuse increases the odds and severity of SCZ [14,16,25]. Although these findings are slightly controversial compared to other studies, a study on ChT found a significant link between physical and sexual abuse with the severity of positive psychotic symptoms in minority ethnic groups [9].

There is strong evidence between ChT and hallucination and delusions [13]. A couple of researchers propose that symptoms such as hallucinations and delusions are intrusions or an individual's perception of intrusions resulting in despair. ChT has a more significant link to positive symptoms than negative symptoms. Emotional neglect is related to paranoia by an individual's belief in a just world. A high perception of injustice and feelings of being a specific target of unfair treatment can cause an individual to be paranoid [24,25].

Comparing reports of ChT between individuals with first-episode psychosis and schizophrenia, the former shows a prevalence rate of 28%-53% while the latter a rate of 9%-83% [26]. SCZ spectrum disorders are associated with lower premorbid adjustment in those exposed to childhood maltreatment. ACEs contribute to neurodevelopment impairment, unveiling poor premorbid adjustment before the onset of psychosis. When addressing premorbid adjustment, findings are not specific to one type of trauma but cover all different categories of ChT [22]. Generally, patients with psychotic disorders and histories of child abuse have common clinical characteristics, such as a higher hospitalization rate for symptoms, a more relentless course of psychosis, earlier onset of symptoms, more severe episodes, heightened rates of treatment failure and noncompliance, greater likelihoods of mood and behavioral disturbances alongside psychosis symptoms, greater risk of suicide and substance disorders [17].

Consideration of trauma histories is an essential factor when studying experiences of psychosis. Children with histories of physical or psychological trauma during periods of personality development in infancy and early childhood years are prone to anxiety and panic episodes in adult years. Panic attacks occur in 45% of patients with SCZ leading to the speculation that early trauma increases the risk of both disorders. A trauma-based approach is necessary when evaluating patients with psychosis since an increase in frequency and severity of ChT correlates with paranoia [19,25,27].

Assessment Tools

Several assessment tools help identify ChT. They take into account severity, context, and cultural influences. The Childhood Trauma Questionnaire (CTQ) is most common across the board, both in non-clinical and clinical individuals [31]. CTQ is an international assessment tool used to assess ChT. This self-report survey, along with other measures, is essential in identifying experiences of abuse and neglect [9]. Most observational studies utilize the Positive and Negative Syndrome Scale (PANSS) to assess psychotic positive and negative symptoms [32]. Additional assessment tools are available to fully explore psychotic traits and symptoms of other mental disabilities. Table 2 contains a summary of necessary assessment tools relating to ACEs and mental health issues.

**Table 2:** Analysis of indicated assessment tools PTSD: post-traumatic stress disorder, CTQ: Childhood Trauma Questionnaire, CTQSF: Childhood Trauma Questionnaire Short Form, DSM: Diagnostic and Statistical Manual of Mental Disorders, OCD: obsessive-compulsive disorder

Assessment tool	Specifics of the assessment tool
Clinician-Administered PTSD Scale (CAPS)	Structured clinical interview with 30 items used as a gold standard for assessing PTSD and quantifying symptom severity [33,34].
CTQ	A retrospective method of measuring childhood trauma using a 70-item questionnaire. It addresses five subscales of trauma: physical neglect, emotional neglect, physical abuse, emotional abuse, and sexual abuse [35,36].
CTQSF	A shortened version of the original CTQ. It uses a 28-item questionnaire [36].
The Chinese version of CTQSF (Childhood Abuse Questionnaire)	A Chinese translated version of the CTQSF, used in the Chinese population due to similar psychometric properties and cultural equivalence [31].
The Dutch version of CTQSF	The self-report CTQSF questionnaire in Dutch [21].
The Childhood Experience of Care and Abuse Questionnaire (CECA.Q)	A self-report questionnaire for obtaining information about incidents of parental hostility, neglects, and abuse [37].
Early Trauma Inventory Self Report - Short Form (ETISR-SF)	A self-administered questionnaire with 27 items to assess general traumatic experiences and childhood trauma occurring before age 18 [38].
Experience in Close Relationships - Relationship Structures (ECR-RS)	A scale to measure attachment within a relational context like family and friends. It can be used on children and adolescents [39].
Liebowitz Social Anxiety Scale (LSAS)	This assessment scale helps measure the severity of social anxiety symptoms. It also measures response to treatment. The scale comprises 24 social situations that rate levels of fear and avoidance. There are two types available: a clinically administered and the other, a self-report version [40].
Neurological Evaluation Scale (NES)	A structured clinical examination helps assess the degree of neurological impairment in schizophrenic patients. The scale comprises 26 items [41].
Panic and Schizophrenia Interview (PaSI)	Interview for assessing comorbidities of anxiety disorder [19].
Positive and Negative Syndrome Scale (PANSS)	The gold standard for evaluating the efficacy of antipsychotic therapy. Evaluation of multidimensional symptoms is done by obtaining data from clinical observations and patient/caregiver reports [42].
Premorbid Adjustment Scale (PAS)	This scale helps assess levels of functioning across four developmental periods from childhood to early adulthood. Areas addressed by the scale include adaptation to school, peer relationships, school performance, social sexual aspect, and sociality [22].
Retrospective Bullying Questionnaire	The questionnaire is helpful in the measurement of victimization during childhood years, assessing physical, verbal, and indirect forms of bullying in school and perceived severity as well as the frequency of bullying [25].
Structured Clinical Interview for DSM-IV (SCID)	An assessment tool widely used to evaluate the presence of anxiety and related disorders [43].
The Avon Longitudinal Study of Parents and Children (ALSPAC)	A prospective cohort study based on a population in the geographical area of Avon in the UK. Pregnant women recruited in 1990-1992 were studied along with their partners and children, since the initial study in 1990 till date [44,45].
The Diagnostic Interview for Psychosis and Affective Disorders (DI-PAD)	An interview using DSM algorithms and the International Classification of Diseases to evaluate symptoms related to depression, mania, and schizophrenia [19].
General Beliefs in a Just World Scale	A six-item, six-point scale, for measuring an individual's belief in a just world in general [25].
MINI International Neuropsychiatric Interview (MINI)	An interview to evaluate psychiatric disorders based on DSM criteria. It measures current and lifetime evidence of substance use, major depression, and psychotic disorders [24].
Personal Belief in a Just World Scale	A seven-item assessment scale with six points; effectively measures individuals' belief that the world is a just place for them [25].
Traumatic Events Inventory (TEI)	A 14-item measurement scale helps screen for a lifetime history of exposure to trauma. It considers the age of first exposure, frequency of traumatic events, and attestation of exposure [46].
Yale-Brown Obsessions and Compulsions Scale (Y-BOCS)	A gold standard assessment scale for severity of symptom evaluation in OCD, widely used by trained clinicians. It consists of two interrelated components: a checklist of obsessions and compulsions, and a severity scale assessing the severity of symptoms during the prior week [47].

Genetics

Generally, people with SCZ have higher peripheral FKBP5 expression; FKBP5 is a gene related to stress response in the hypothalamic-pituitary-adrenal (HPA) axis. Notably, the FKBP5 gene functions in developing psychosis and the outcome of SCZ amidst exposure to acute or chronic stress. The role of various FKBP5 gene variants can be beneficial or detrimental when considering the risk of illness, worsening of psychosis, and response to therapy. Increased cortisol secretion in schizophrenic patients with ChT theorizes that the HPA axis dysfunction in SCZ is related to ChT [15,26].

Research has also shown that the genetic factor brain-derived neurotrophic factor (BDNF) Val66Met polymorphism directly connects to SCZ symptoms, in addition to the interplay of genes and environmental factors, especially ChT. When exposed to ChT, BDNF Met carriers have increased positive psychotic experiences compared to their counterparts [20].

The loss of volume in at-risk brain regions such as the hippocampus is a common finding in SCZ. Low hippocampus BDNF levels are present in animal models exposed to acute and chronic stress using glucocorticoids as a stress inducer. Cortisol use in medical treatment can provoke psychotic symptoms. Although recent meta-analysis reports show a desensitized cortisol response to stress in SCZ, SCZ patients have a flattened reaction to stress and lower thresholds than controls during and after stress exposure validating that SCZ patients have a substantial impairment in their stress response. Interestingly, adolescents at high risk of psychosis have high resting cortisol levels related to environmental influences such as negative self-concept and emotional abuse from relatives. Still, when these individuals develop symptoms, their stress response becomes significantly desensitized [18].

Genetic liability for SCZ is associated with specific environmental trauma exposure; individuals at high risk would benefit from more significant support and resources to promote a healthier developmental environment [16].

Neural Pathways and Neuropathophysiology

There is a correlation between histories of child maltreatment with several structural changes in the brain. Imaging studies show a decreased volume of the hippocampus, dorsolateral prefrontal cortex, amygdala, cerebellum, inferior frontal gyrus, gray matter, and anterior cingulate cortex in patients with ACEs and psychotic disorders. The thalamus is also majorly affected in these patients; dysfunction in connectivity of the thalamus and different brain regions is a strong predictor of conversion of prodromal syndromes to full-blown psychotic disorders in at-risk adults and adolescents. A study examining the influence of early trauma on emotional processing found vital results in SCZ patients with histories of sexual abuse: decreased functional connectivity between the sulci of the left precuneus, left posterior cingulate, and right calcarine and amygdala predominantly in this patient group [17].

The HPA axis invariably shows alteration by ChT. Individuals with histories of childhood abuse show hyperactivity of the axis leading to subsequent alterations in dopamine, the autonomic nervous system, brain structure, and neural function, which all increase the probability of susceptibility to psychosis. The elevation of striatal dopamine function is evident in those exposed to childhood adversity, connecting psychogenic stress, especially sexual and physical abuse, to associative striatum elevation [23,24].

In 2001, after studying the positive symptoms of psychosis, a cognitive model elaborated that predisposition to develop psychosis has a bio-psycho-social origin. Since childhood, traumatic experiences can lead individuals to have a faulty view of themselves, and they also construct a fear-based outlook of the world; suspicion, intrusive thoughts, dissociation, and paranoia can very well become coping mechanisms. Although this is not entirely accepted, these findings are sufficient to explain the role of trauma and psychosis in specific individuals [27].

As noted earlier, the hypothesis of SCZ pathophysiology mainly lies in dopamine overstimulating the D2 receptors in the brain. Studies propose stress sensitization as a critical part of SCZ decreasing the sensitivity threshold in people with SCZ. Stress can activate the HPA axis, which results in the sensitization of dopamine in the mesolimbic region and an increase in the striatal dopamine release. A meticulous review considering the results from studies on child abuse and psychosis shows that psychosocial stress triggers psychosis through mechanisms of emotional changes and disturbance of cognitive processing in at-risk individuals [18,19].

Psychopathological symptoms strongly reconcile the link between ChT and psychosis; anxiety can trigger the development of paranoia, a false sense of reality, and, in the long run, hallucinations. One trauma can also accentuate the effects of another existing traumatic experience leading to a faster pathway to symptomatic presentation [21].

Limitations

The empirical findings reported herein should be considered in light of some limitations. Firstly, the preferential selection of articles published in English or fully translated and free full-text articles led to the exclusion of plausible relevant findings. Additionally, some individuals exposed to extreme ACEs are not affected by psychosis later in life, a concept unaddressed in this study; this exception is worth exploring with further research.

Finally, this review has no significant analysis of confounders and modifiers in the development of psychosis/SCZ, such as substance use, family history, personality disorders, low birth weight, and neonatal infection exposure. Therefore, a cautious interpretation of findings from this study is necessary when considering confounding variables. In the future, a study addressing the interaction between various confounding variables present in schizophrenic individuals could provide additional insights into the pathogenesis and prevention of SCZ.

## Conclusions

This systematic review adds to the currently available data, acknowledging that adversity faced during infancy through teenage years can manifest in adulthood. Lately, the recognition of individual lived experiences has shown positive impacts on the management of several psychiatric disorders. This study's primary purpose is to emphasize the importance of evaluating patients as a whole, acknowledging the effects of their previous experiences in the manifestation of current symptoms. Evidence suggests that neurobiological processes occur in the brain after trauma, predisposing individuals to developing psychosis and SCZ. The evaluation of the benefit of initial trauma detection and the availability of early support is critical in future trials to highlight the feasible potential of neurological effect modulation. Identifying a positive impact on neurobiology should encourage more primary care providers to screen for trauma early on as part of child wellness visits. In ideal circumstances, early detection of adversity in individuals will lead to an immediate introduction of available resources beneficial in combating mental and emotional dysregulations, a possible key to decreasing the diagnosis per year and intensity of schizophrenia in the general population.
